# BERTrand—peptide:TCR binding prediction using Bidirectional Encoder Representations from Transformers augmented with random TCR pairing

**DOI:** 10.1093/bioinformatics/btad468

**Published:** 2023-08-03

**Authors:** Alexander Myronov, Giovanni Mazzocco, Paulina Król, Dariusz Plewczynski

**Affiliations:** Faculty of Mathematics and Information Science, Warsaw University of Technology, Warsaw, Poland; Ardigen, Krakow, Poland; Ardigen, Krakow, Poland; Ardigen, Krakow, Poland; Faculty of Mathematics and Information Science, Warsaw University of Technology, Warsaw, Poland

## Abstract

**Motivation:**

The advent of T-cell receptor (TCR) sequencing experiments allowed for a significant increase in the amount of peptide:TCR binding data available and a number of machine-learning models appeared in recent years. High-quality prediction models for a fixed epitope sequence are feasible, provided enough known binding TCR sequences are available. However, their performance drops significantly for previously unseen peptides.

**Results:**

We prepare the dataset of known peptide:TCR binders and augment it with negative decoys created using healthy donors’ T-cell repertoires. We employ deep learning methods commonly applied in Natural Language Processing to train part a peptide:TCR binding model with a degree of cross-peptide generalization (0.69 AUROC). We demonstrate that BERTrand outperforms the published methods when evaluated on peptide sequences not used during model training.

**Availability and implementation:**

The datasets and the code for model training are available at https://github.com/SFGLab/bertrand.

## 1 Introduction

Cytotoxic T-cells play a major role in adaptive immune response in humans. Intracellular proteins are degraded by proteasome into peptides. The antigen processing machinery of the cell allows the presentation of peptides on the cell surface using Major Histocompatibility Complexes (MHC). The focus of this work is MHC class I, which typically presents a peptide of 8–11 amino acids long. These peptide-MHC (pMHC) complexes in turn can be recognized and engaged by CD8+ cytotoxic T-cells. Due to negative selection in the thymus and the high degree diversity of the T-cell receptor (TCR) repertoire, T-cells are capable of recognizing a variety of foreign and mutated epitopes (Rudolph *et al.* 2006).

The binding properties of a TCR to a given pMHC is regulated by hypervariable Complementarity Determining Regions (CDRs). For the *α* and *β* chains of the TCR, three such regions exist. The CDR1 and CDR2 of both *α* and *β* chains mostly interact with the MHC complex, while CDR3 is predominantly interacting with the peptide. The CDR3 region is the most variable region of the TCR and is thought to be the major factor in determining the binding preference of the TCR toward its conjugated pMHC (La Gruta *et al.* 2018). While both *α* and *β* chains contribute to the interaction, some studies suggest that the *β* chain plays a more important role in antigen recognition (Sidhom *et al.* 2021) and is significantly more prevalent in the data. It should be noted that many researchers—Dash *et al.* (2017) and Montemurro *et al.* (2021)—have demonstrated the importance of the *α* chain of the TCR in antigen recognition. However, this work implies the use of machine learning (ML) to infer the interaction between TCRs and pMHC complexes, so it can benefit from a high number of observations. In the data we collected, only 18% of the observations have the CDR3*α* annotation. Thus, we will be focusing solely on the sequence of the CDR3*β* part of the TCR, which is readily available in multiple datasets. This is the approach also adopted by Weber *et al.* (2021) and Lu *et al.* (2021).

The MHC is not able to present each and every peptide. Thanks to a high amount of pMHC binding data as well as peptide presentation data from mass spectrometry experiments, researchers were able to produce high accuracy models of pMHC binding and presentation. However, even if the peptide is presented on the surface of the cell, it is still unlikely to be immunogenic. The study by Parkhurst *et al.* (2019) of peptide T-cell immunogenicity for 75 cancer patients has been able to find only 57 CD8+ positive mutations along with over 8000 non-immunogenic ones, which account for <1%. One of the frontiers of computational immunology research currently is peptide:TCR binding prediction, which represents a key component for understanding T-cell activation. The amount of data for peptide:TCR binding prediction has been growing in recent years, with the popularization of pMHC dextramer production, single-cell TCR sequencing, and TCR barcoding. In this work, we compile a collection of peptide:TCR sequence data from a number of databases and publications into a single curated dataset of known TCR binders with their cognate epitope sequences. We augment the dataset with negative decoy examples generated from reference T-cell repertoires.

The growing amount of data on peptide:TCR specificity allowed for the creation of many computational tools that facilitate peptide:TCR binding prediction. These tools can be broadly categorized into three groups. The first group of methods uses the similarity between TCRs to produce clusters and determine their peptide specificity. GLIPH (Glanville *et al.* 2017) and TCRdist (Dash *et al.* 2017) have demonstrated that for a given epitope sequence, TCR binding can be predicted accurately using distance-based methods. The second group of methods involves the training of peptide-specific TCR binding models—DeepTCR (Sidhom *et al.* 2021), TCRex (Gielis *et al.* 2019), and TCRGP (Jokinen *et al.* 2021). These algorithms often work remarkably well for known peptides but are unable to predict binding for unseen peptides. The third group of methods are the methods that allow prediction for unseen peptides—NetTCR2.0 (Montemurro *et al.* 2021), ERGO (Springer *et al.* 2020), pMTnet (Lu *et al.* 2021), DLpTCR (Xu *et al.* 2021), TITAN (Weber *et al.* 2021), and PanPEP (Gao *et al.* 2023). The goal of this research is to provide immunologists with better tools for *in silico* TCR therapy design. As most of the peptides in the published data originate from viruses, peptide-centric models from the second group have limited applicability for cancer neoantigens or tumor-associated antigens. The focus of this work is thus the peptide:TCR pairing task, specifically the case when the model has not previously seen neither the peptide nor the TCR. We believe that high accuracy for this task would bring the most benefit for potential users.

Recent breakthroughs in Natural Language Processing (NLP), such as TAPE (Rao *et al.* 2019) and DNABERT (Ji *et al.* 2021), have prompted many researchers to apply Transformer architectures to solve sequence-based biological problems, such as transcription factors prediction, protein–protein interaction prediction, and binding pockets prediction to name a few. One useful feature of models from the NLP domain is the ability to process variable-length sequences of symbols from a fixed alphabet. Another important aspect is the ability to benefit from unsupervised pre-training, which is often an option in bioinformatics. Researchers usually pre-train the language model on large sequence databases, such as UniPROT. In this work, we construct a pre-training set for the peptide:TCR model, which comprises a hypothetical human peptide:TCR repertoire, based on peptides from MHC-I mass spectrometry peptide presentation experiments and TCRs from healthy donors. After pre-training our model is fine-tuned to predict peptide:TCR binding and was shown to outperform the existing methods in cross-peptide generalization task.

**Figure 1.**
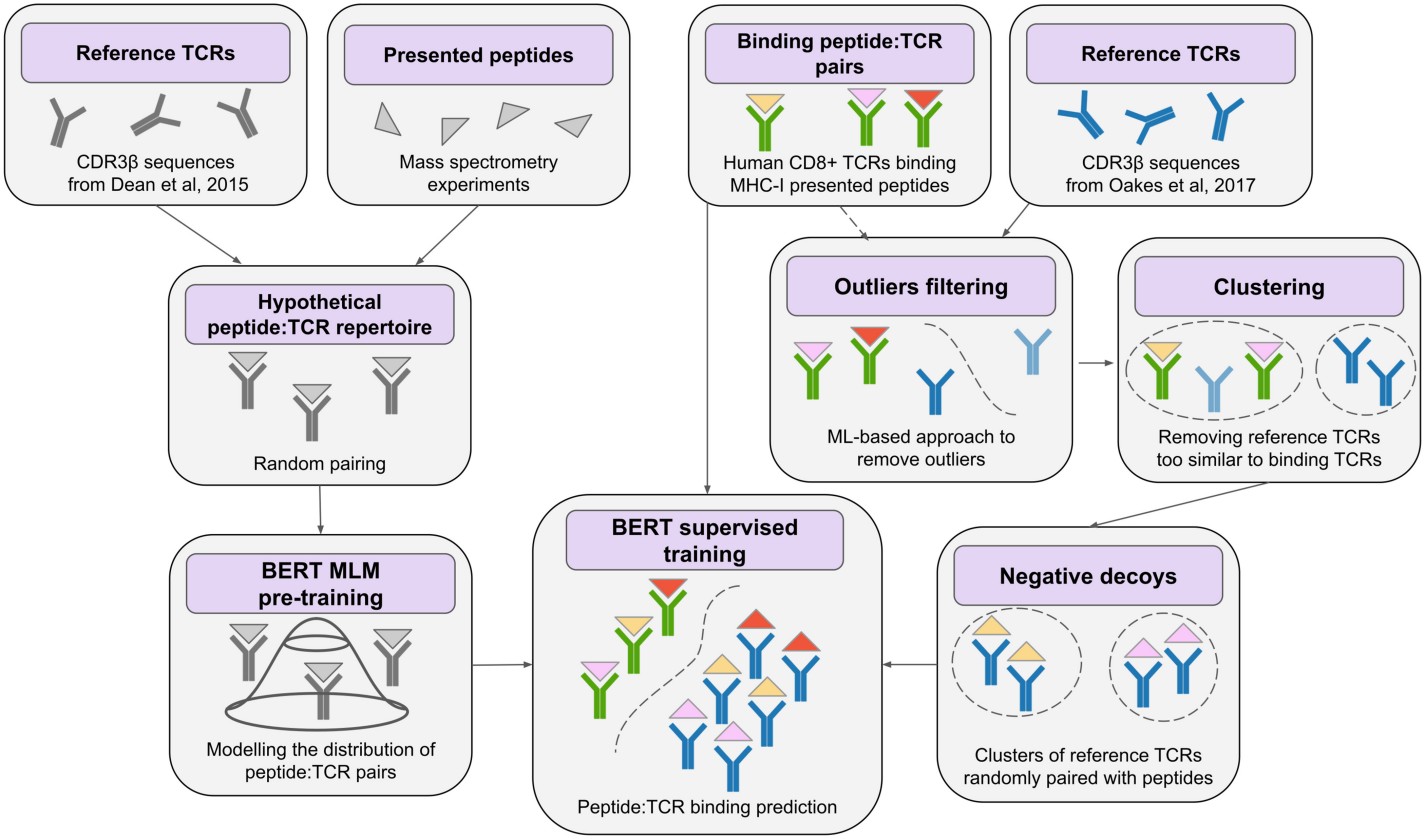
Flow diagram of the analysis. The left part illustrates the creation of the hypothetical peptide:TCR repertoire and MLM pre-training. The right part shows the process of negative decoys generation and various filtering steps leading to it. These two paths converge on BERT supervised training for peptide:TCR binding prediction

The overall flow of the analysis is demonstrated in Fig. 1. In the left part of the figure, we show the process of the NLP model pre-training. Reference TCR sequences from healthy donors are paired randomly with the presented peptides to produce a hypothetical peptide:TCR repertoire, which is then used to perform masked language modeling (MLM) pre-training of the Bidirectional Encoder Representations from Transformers (BERT) neural network. In the right part of the figure, the process of generating negative decoy observations is illustrated. Negative decoys are also based on reference TCRs. A ML model is used to remove outliers, and then the remaining reference TCRs are clustered together with binding TCRs. TCR sequences that are too similar to any binding TCR are removed, and the rest of the reference TCR clusters are randomly paired with peptides from the binding peptide:TCR set. Pre-trained BERT network is then trained to predict peptide:TCR binding.

## 2 Materials and methods

### 2.1 Data

#### 2.1.1 Data curation

**Figure 2.**
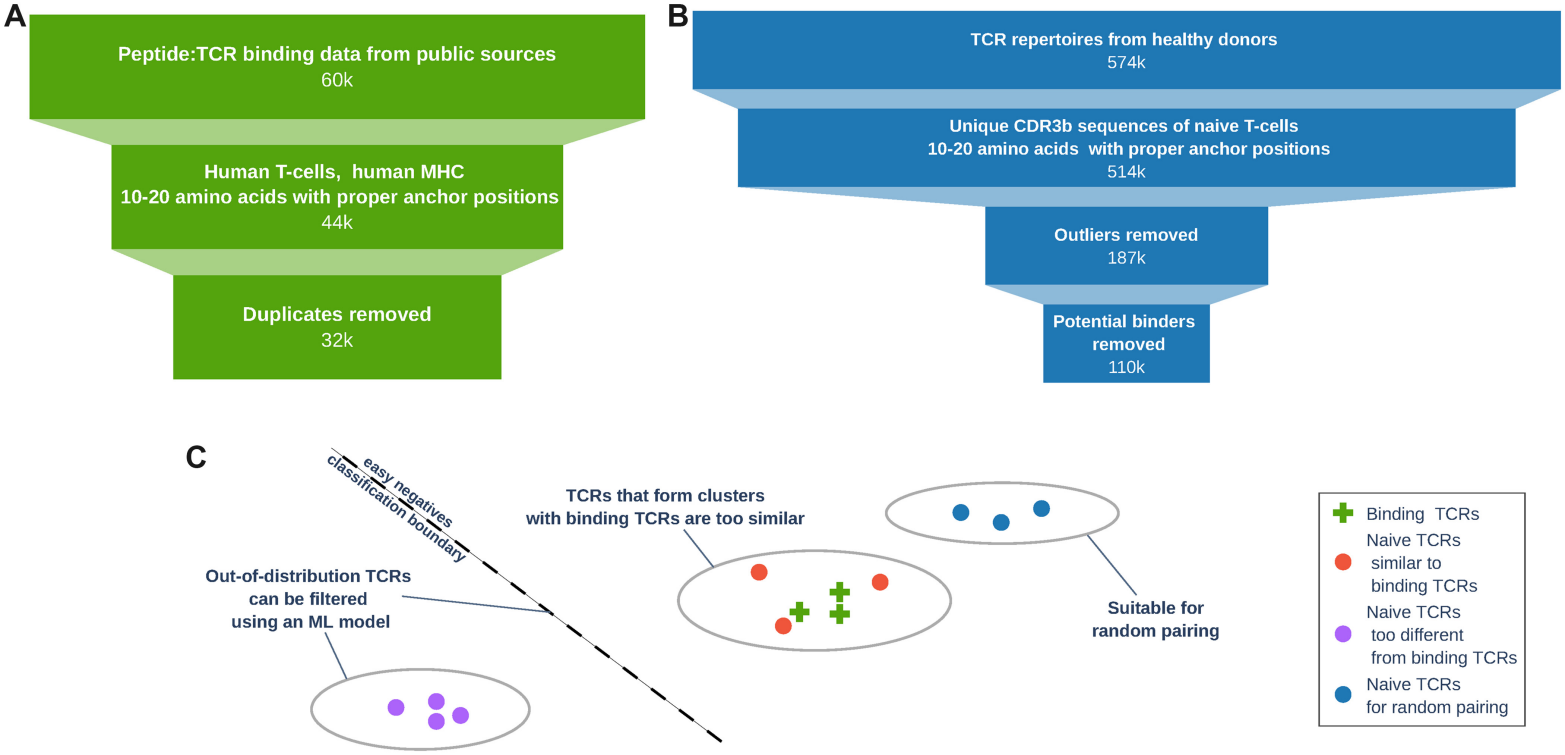
Data curation process. (A) Binding peptide:TCR observations. (B) Reference TCRs for decoy generation. (C) Reference TCRs filtering illustrated

Our data curation process is shown in Fig. 2. We collected data containing the amino acid sequences of binding peptide:TCR pairs from a number of databases and publications (Table 1). We narrowed down the dataset to human CD8+ T-cells for peptides of 8–11 amino acids long. We only considered CDR3*β* observations, as CDR3*α* annotations are available for only 18% of the data (<6k observations). Over 99% of the CDR3*β* sequences have a length between 10 and 20 amino acids. Another filtering criterion was the requirement of having specific amino acids in the first and last anchor positions in CDR3*β* chains, cysteine (C) and phenylalanine (F), respectively. This way, we compiled 33k unique CDR3*β* sequences of T-cells binding with a total of 401 epitope sequences. To compensate for the lack of negative examples, we generated negative decoy observations in a 3-to-1 ratio, using a dataset of reference T-cell repertoires from healthy donors (Oakes *et al.* 2017) and paired it randomly with peptides from the binders dataset.

**Table 1.** Summary of the binding peptide:TCR dataset.^a^

Dataset	Number of observations
VDJdb (Shugay *et al.* 2018)	15 751
McPAS (Tickotsky *et al.* 2017)	8482
Pogorelyy *et al.* (2018)	5861
TBAdb (Zhang *et al.* 2020)	5852
10x Genomics (Boutet *et al.* 2019, Zhang *et al.* 2021)	3813
Zhang *et al.* (2018)	1093
Huth *et al.* (2019)	1039
Emerson *et al.* (2017)	803
Abdel-Hakeem *et al.* (2017)	324
Britanova *et al.* (2016)	260
Kamga *et al.* (2019)	217
Gee *et al.* (2018)	33
Takeda *et al.* (2018)	20
Soon *et al.* (2019)	6
Malekzadeh *et al.* (2019)	4
Total	43 558
Unique	32 523

a The number of observations is reported after filtering. The curated set of unique peptide:TCR binders is later used to train the model.

**Table 2.** The overview of potential biases that a ML algorithm for peptide:TCR prediction can exploit.

Bias	Mismatch pairing	Reference pairing
Under-representation of peptides in the dataset	Yes	Yes, but it is mitigated by pre-training to some extent
Under-representation of TCRs in the dataset	Yes	No, because additional TCR sequences are used
Potential false negatives	Yes	Yes
Out-of-distribution easy negative decoys	No	Yes, but they can be filtered to some extent
Too homogeneous negative decoys	Yes	No
Correlated observations in train and test sets	Yes, because mismatch pairing introduces additional dependencies	No, peptide and TCR clusters are used for decoy generation and cross-validation

Cluster analysis of the peptides was done using hierarchical clustering with Levenshtein distance and single linkage. It revealed that a number of similar epitope sequences are present, differing by three amino acids at most. For example, MART-1 human melanoma-related antigen (ELAGIGILTV) was extensively studied with minor modifications: EAAGIGILTV, LLLGIGILV, ELAGIGLTV, AAGIGILTV, and ALGIGILTV. We argue that using such similar observations in the training and validation set may bias any model trained on peptide sequences and could introduce unwanted overfitting (if the TCR repertoires of two similar peptides are also similar), or unwanted underfitting if the repertoires are too different. Our analysis revealed 261 peptide clusters with a minimum Levenshtein distance of three. Peptide clusters were used as groups during cross-validation.

#### 2.1.2 Sources of overfitting

Training a ML model on a limited set of data (i.e. positive peptide:TCR pairs for 400 peptides only) and with a lack of negative examples forces us to create negative decoy observations, which may easily introduce some bias that an ML model could easily exploit. Randomly pairing peptides and TCRs to create negative decoys is certainly a viable approach for this problem. TCRs are highly cross-reactive and are estimated to bind 10^6^ multiple epitope sequences (Mason, 1998), hence a TCR could potentially bind some other peptide. However, TCRs are also highly specific, as the probability of a specific TCR binding a randomly chosen peptide is estimated to be $10^{-4}$ (Frank, 2020), which is acceptable for a ML approach. Besides possible false negative observations produced by random pairing and biases that may arise from experimental conditions, we have identified a handful of potential problems for a training setup with negative decoys (see Table 2).

#### 2.1.3 Mismatch pairing

Mismatch pairing is an approach used in Montemurro *et al.* (2021) and Springer *et al.* (2020) to create negative decoy observations by randomly pairing a peptide with a TCRs from a different peptide:TCR pair. Mismatch pairing approach guarantees that the TCR distribution of the negatives will match the positive one. The number of different TCRs in a human body is estimated to be around $10^{8}-10^{10}$ (Qi *et al.* 2014, Lythe *et al.* 2016), and the number of MHC-I-presented peptides on human cells is around 10^4^ (Mester *et al.* 2011), thus mismatch pairing is largely under-representing both peptides and TCRs. Moreover, due to publication bias, different potential immunogenicity of viral and cancer peptides in humans, the distribution of T-cell clonotype size in humans (Bolkhovskaya *et al.* 2014) and other factors, the number of unique TCR observations per peptide has a power-law decay distribution (see Supplementary Fig. S1). In practice, this means that random peptide:TCR pairing will produce negative decoys, where over 61% of TCR sequences come from the five most popular peptides in the dataset. The repertoires of these peptides are too homogeneous and a model might learn to identify those as negatives. Mismatch pairing also produces correlated observations, thus during cross-validation there would be a considerable amount of examples in the training set sharing peptide or TCR sequence with pairs in the test set. These recurring peptides and TCRs may be exploited by the model if not removed. Deep learning models have a tendency to “remember” training data, so correlated observations in the training and test sets should be avoided to ensure test set independence.

#### 2.1.4 Reference pairing

Our approach to negative decoys generation was designed to overcome some of the aforementioned biases. We collected around 560k TCR CDR3*β* sequences from repertoires of three healthy donors from Oakes *et al.* (2017) and randomly paired them with peptides from the binding dataset CDR3*β* sequences. The study by Oakes *et al.* (2017) was used for reference pairing instead of Dean *et al.* (2015) due to the improved sequencing protocol that captured the rare clones in the TCR distribution, allowing for a more biologically plausible CDR3*β* sequence space, and the availability of the T-cell type annotation. We used only the CD8+ T-cells from the patients’ repertoires, which matches the MHC class I peptides in the binders’ dataset. Reference TCRs represent a much larger region of the TCR theoretical distribution compared to binding TCRs, although both TCRs and peptides remain still under-represented. Reference TCRs obtained through a different kind of TCR sequencing experiment might introduce CDR3*β* sequences that are out-of-distribution relative to the binding TCRs and thus might be easy targets for a neural network. To address this issue, we filtered the sequences using a ML-based approach, see Outliers filtering section in the Supplementary Material for a detailed description.

To avoid correlated observations, we performed TCR clustering analysis of the binding TCR sequences together with reference TCRs: hierarchical clustering was used with Levenshtein distance and complete linkage with distance cut-off equal to three. Clustering naturally produced three types of clusters:

1. clusters with only TCRs from binding peptide:TCR pairs
2. mixed clusters positive and reference TCRs
3. clusters with only reference TCRs.

During decoy generation, we rejected reference TCRs from mixed clusters, as they have a much higher probability of being false negatives, because their sequences are very similar to those of the binding TCRs. TCRs from reference-only clusters were randomly paired with a single peptide from the pool of 401 available peptides in the binding dataset in a 3-to-1 ratio to the number of positive observations for a given peptide. In a cluster of reference TCRs, every TCR is paired with the same peptide, which limits the generation of positive and negative observations with the same peptide sequence and very similar TCR sequences. Such observations would in fact be quite useless, as they should be filtered from the training set if they appear in the test set during cross-validation, otherwise the model would be biased toward predicting the negative class. As the decoy generation is random, the dataset was replicated three times for different seeds. TCR clusters were also used as groups during cross-validation.

### 2.2 Model

For this problem, we applied the BERT artificial neural network (Devlin *et al.* 2019) from “transformers” python package (Wolf *et al.* 2019). The model was initially pre-trained to perform a MLM task on a hypothetical TCR repertoire and then fine-tuned for actual peptide:TCR classification.

#### 2.2.1 Model architecture

**Figure 3.**
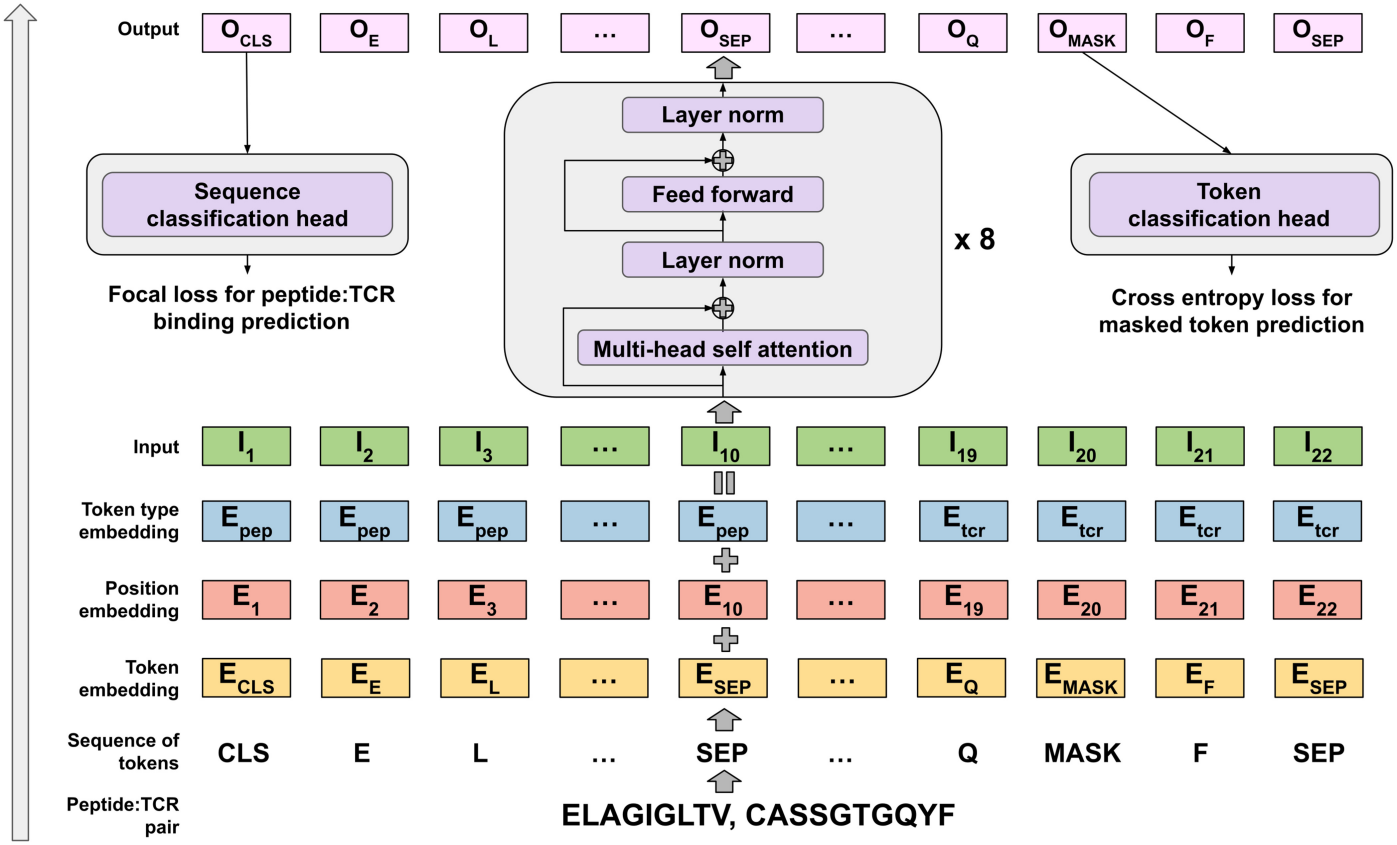
BERT architecture (bottom to top): first, peptide and TCR are tokenized. Then, each token is embedded in a 512-dimensional token space. The absolute position and token type—peptide or TCR—are encoded using separate embeddings. All three embeddings are added to form the input. Eight transformer blocks process the input to produce the output for each token. During MLM pre-training, the token classification head (right) is trained to predict the true token for 15% randomly masked tokens. During sequence classification training (left), the sequence classification head takes output of the CLS token and predicts binding for a peptide:TCR pair

The architecture of BERT is illustrated in Fig. 3. Initially, peptides and TCRs need to be represented as a sequence of tokens. The token vocabulary consists of 20 amino acids and 5 additional special tokens: CLS is used to indicate the starting position of the sequence, SEP to indicate the end of the sequence, MASK for MLM, PAD for padding, and UNK for non-standard amino acids. Each peptide and TCR are concatenated into a single sequence of tokens with an additional CLS token at the beginning and two SEP tokens between sequences and at the end of the sequence. The sequence IDs indicating the absolute position of the token and the token type IDs, indicating whether a token belongs to the peptide or to the TCR, are generated as well. Token, position and type IDs are embedded and added creating the sequence of input embeddings for each token. The input is then passed to a BERT network with eight transformer blocks. BERT produces an output embedding for each token, which later on is passed to either a token classification head and to a sequence classification head during pre-training and fine-tuning, respectively. Below is the description of the BERT embedding procedure.


$$\begin{array}{c} \text{token}\_\text{ids}=[\text{CLS}]+\mathrm{tokenize}(\text{Peptide})+[\text{SEP}]+\\ +\mathrm{tokenize}(\text{CDR}3\beta )+[\text{SEP}]\\ \text{position}\_\text{ids}=[1,2,3,\ldots ,\mathrm{length}(\text{Peptide})+\mathrm{length}(\text{CDR}3\beta )+3]\\ \text{token}\_\text{type}\_\text{ids}=[\underset{\mathrm{length}(\text{Peptide})+1}{\underset{︸}{1,1,1,\ldots ,1}},\underset{\mathrm{length}(\text{CDR}3\beta )+1}{\underset{︸}{2,2,2,\ldots ,2}}]\\ \text{embeddings}=\mathrm{BERT}(\text{token}\_\text{ids},\text{token}\_\text{type}\_\text{ids},\text{position}\_\text{ids}). \end{array}$$

#### 2.2.2 Pre-training

The large (over 2.5 M) number of trainable parameters in BERT is compensated by the unsupervised pre-training strategy. For this purpose, we created the hypothetical peptide:TCR repertoire. For CDR3*β* sequences, we used the TCR repertoires of 85 healthy individuals from a large high-throughput immunosequencing study by Dean *et al.* (2015), which resulted in 11 M sequences. Over 150k peptides were acquired from the results of mass spectrometry peptide sequencing experiments (Abelin *et al.* 2017, Di Marco *et al.* 2017, Faridi *et al.* 2018, Sarkizova *et al.* 2020). We randomly paired the MHC-I-presented peptides with reference TCRs and pre-trained a BERT neural network using MLM. Fifteen percentage of randomly chosen amino acids in the input sequence were masked and the network was trained to predict the masked amino acid. The weights from this stage were used as a starting point for the supervised training task. The effect of MLM pre-training can be seen in Supplementary Fig. S2. The network was pre-trained for 100 epochs on a dataset of 9 M peptide:TCR pairs, 2 M of peptide:TCR pairs were used for validation. See Hyperparameters section in the Supplementary Material for more details about model training. Below is the description of the BERT MLM procedure.


$$\begin{array}{c} p=\mathrm{Bernoulli}(0.15)\\ \text{masked}\_\text{token}\_{\text{id}}_{i}=\{\begin{array}{cc} \text{MASK} & \text{if}\;p=1,\\ \text{token}\_{\text{id}}_{i} & \text{if}\;p=0. \end{array}\\ \text{embeddings}=\mathrm{BERT}(\text{masked}\_\text{token}\_\text{ids},\text{token}\_\text{type}\_\text{ids},\text{position}\_\text{ids})\\ \text{predicted}\_\text{token}\_\text{ids}=\mathrm{token}\;\mathrm{classification}\;\mathrm{head}(\text{embeddings})\\ \text{MLM loss}=\mathrm{crossentropy}(\text{token}\_\text{ids},\text{predicted}\_\text{token}\_\text{ids}). \end{array}$$

#### 2.2.3 Fine-tuning

A separate sequence classification head was trained to classify binding peptide:TCR pairs and negative decoys. The hidden representation of the first token in the sequence was passed into a feed-forward layer combined with a softmax layer output. Focal loss with γ = 3 and α=0.25 was used. Below is the description of the BERT fine-tuning procedure.


$$\begin{array}{c} \text{embeddings}=\mathrm{BERT}(\text{token}\_\text{ids},\text{token}\_\text{type}\_\text{ids},\text{position}\_\text{ids})\\ \text{embedding}\_\text{CLS}={\text{embeddings}}_{0}\\ \hat{y}=\mathrm{sequence}\;\mathrm{classification}\;\mathrm{head}(\text{embedding}\_\text{CLS})\\ \text{supervised loss}=\mathrm{focal}\;\mathrm{loss}(y,\hat{y},\gamma =3,\alpha =0.25). \end{array}$$

### 2.3 Benchmarks and evaluation

Existing approaches to peptide:TCR binding predictions based on peptide and CDR3*β* sequences were evaluated alongside our model. The selection criteria for the benchmarks were the following—ability for peptide:TCR binding prediction for unseen peptides, code availability with the possibility of re-training the model on our dataset, and the ability to be trained without CDR3*α* chain information. Among the peptide:TCR binding methods mentioned earlier, four algorithms fulfill the above criteria, namely NetTCR2.0, ERGO, TITAN, and DLpTCR. NetTCR2.0 was published by Montemurro *et al.* (2021), which uses a convolutional neural network (CNN) to predict peptide:TCR binding for A02:01-restricted peptides. ERGO is the algorithm published by Springer *et al.* (2020), which uses a pre-trained long short-term memory neural network (LSTM) architecture. TITAN from Weber *et al.* (2021) is a bimodal neural network, which is pre-trained on general protein–ligand interactions and uses an atomic-level SMILES input for peptide that is combined with a TCR input using cross-attention. DLpTCR from Xu *et al.* (2021) uses an ensemble of CNNs, LSTMs and fully connected neural networks. We are grateful to the authors of these approaches for providing the full source code for model training.

To test the generalization power of our model and the benchmarks, we performed repeated cross-validation grouped by peptide and TCR clusters, to avoid correlated observations in the training and testing set, which can cause inflated results due to neural networks’ ability to remember training examples. For each training episode, the dataset was split into train and test sets. Fourteen peptide clusters and all their associated TCRs were chosen for each test set. Train observations with a TCR belonging to any test set TCR cluster were removed. All models were trained using the same dataset splits to ensure fairness in terms of data availability. The train and test sets were restricted to viral peptides and the final quality of the predictions was measured on an independent set of 76 cancer peptides. This process was repeated 21 times for 3 repetitions of random pairing, resulting in 63 rounds of cross-validation in total.

The metric used for model validation was AUROC, which is the most popular metric for this problem (Lu *et al.* 2021, Weber *et al.* 2021, Xu *et al.* 2021, Meysman *et al.* 2023). It was computed separately for each peptide and then averaged. Using this averaging procedure, we limit the bias, which originates from the high number of observations for some peptides and from the differences between peptide repertoires. We believe that such a measure is preferable to AUROC computed across all the observations, as the latter might likely lead to inflated results.

Bertrand was trained for 25 epochs. The benchmarks were trained according to the training procedures provided in their corresponding repositories. BERTrand, ERGO, TITAN, and DLpTCR use early stopping on a separate subset of the data, however, NetTCR2.0 used training loss for early stopping. The specifics of the evaluation procedure are explained in detail in the Evaluation section of the Supplementary Material.

Two kinds of baselines were considered during the evaluation: a random baseline (which is equal to 0.5 for AUROC), and baseline based on the prediction using TCR sequence only. The second baseline was estimated by training BERTrand without the peptide sequence. This is a more realistic baseline that represents the biases in the CDR3*β* sequences introduced by the negative decoys generation. For more details about the baselines, see the Baseline estimation section in the Supplementary Material.

## 3 Results and discussion

**Figure 4.**
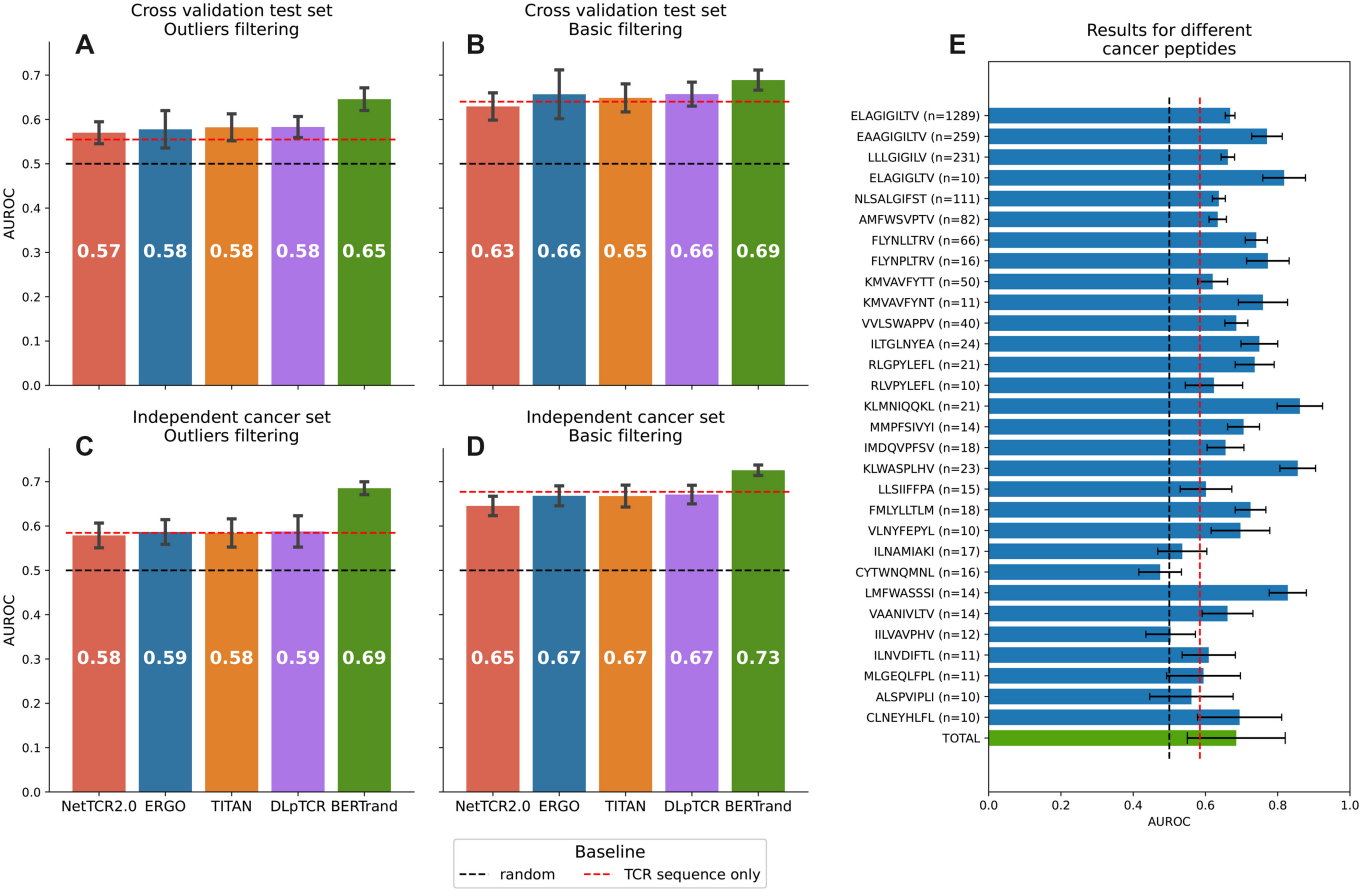
Benchmarks. (A) Results for the cross-validation test set. (B) Results for the cross-validation test set without outliers filtering demonstrate the potential metrics inflation due to outliers. (C) Results for the independent cancer set. BERTrand demonstrates better performance that the other models. (D) Results for the independent cancer set without outliers filtering are inflated and again demonstrate the importance of outliers filtering. (E) Results for individual peptides in the independent cancer set. Note that the confidence interval of the mean AUROC is wider as it includes high per-peptide variation

We performed 21 rounds of cross-validation, repeated three times with different random pairings, and we evaluated the average per-peptide AUROC for each model. BERTrand converged to the optimal solution at around five epochs, with further training only introducing overfitting (see Supplementary Fig. S2). The cross-validation results shown in Fig. 4 indicates that our model can achieve better predictive performance than the state-of-the-art models when tested in multiple scenarios. While the average AUROC indicates a model that is definitely better than the baseline, the high variability in AUROC between peptides is definitely a concern. Out of 76 cancer peptides BERTrand achieved over 0.58 AUROC for 62 of them. While the model may be not optimally suited to perform prediction tasks on single peptide targets, it is applicable to groups of peptide targets in a practical *in silico* TCR prioritization scenario. With a large enough set of previously unseen peptide targets, BERTrand can generate predictions that prioritize binding peptide:TCR pairs, yielding an expected AUROC of 0.69.

We performed additional validation by using a different metric—namely average precision (AP). The AP on the cancer dataset for BERTrand is 0.55, which is better than all four benchmarks. See the Average precision section in the Supplementary Material for these results and the discussion on the metric choice.

Table 3 summarizes the effect of the two most computationally intensive steps of the pipeline—NLP pre-training (12 days using 4 NVIDIA A100 GPUs) and outliers filtering (7 days using 128 CPUs). More detailed results on the convergence of BERTrand without pre-training can be found in the NLP pre-training section of the Supplementary Material.

**Table 3.** Comparison of the results for the independent cancer set without the most computationally intensive steps.

Condition	AUROC	Explanation
Baseline	0.69	All steps in the pipeline
No pre-training	0.62	Poor results due to the a large number of weights trained from scratch
No outliers filtering	0.73	Inflated results due to out-of-distribution reference TCRs

We believe that the biggest challenge in peptide:TCR binding prediction is peptide bias due to low diversity of the peptides available. It is obvious that neither the peptide space (i.e. all possible peptides to be bound by TCRs) nor the TCR space (all possible TCRs) is sufficiently explored in the published data. NetTCR2.0 uses CNNs to predict the binding, which are very susceptible to overfitting when an obvious bias, such as a very popular peptide in the training dataset, is present. ERGO overcomes the TCR diversity problem by applying unsupervised TCR pre-training, but the peptide bias remains unaddressed. TITAN also uses a pre-trained model, but it is pre-trained on general protein–ligand interactions, which may be too different from the peptide:TCR distribution. The strategy of pre-training on a biologically plausible joint peptide:TCR distribution might help to address the peptide bias, as we have shown in this work. Language-based models can be successfully applied to the peptide:TCR binding problem outperforming other state-of-the-art methods, as highlighted in the results of our tests.

## 4 Conclusions

Cross-peptide generalization in peptide:TCR binding prediction remains a hard problem. However, results presented in this work demonstrate that peptide:TCR binding is predictable beyond known peptide targets. We believe the biggest obstacle in this field is data availability. Recent advances in single-cell TCR sequencing allowed for producing more experimental data, so we hope this work will encourage future peptide:TCR binding experiments and in the end, allow predictive models to become very useful for researchers. Potential applications of the peptide:TCR prediction model include the design of off-the-shelf TCR-based therapies for cancer (e.g. TCR-engineered T-cell therapies, TCR-mimicking antibodies, and TCR bispecific antibodies), the development of de-immunization strategies for autoimmune diseases, and the selection of optimal candidates for antiviral vaccine design. Even a model with a limited predictive power can already represent a very useful tool for the optimization of *in vitro* experiments (e.g. reducing the experimental time and costs associated with the typical experimental testing of a large number of putative non-prioritized TCR candidates).

An important limitation of this work is the lack of CDR3*α* due to low availability of those sequences in databases. Although CDR3*α* sequence has been reported to be important for peptide:TCR binding prediction (Sidhom *et al.* 2021), it is only available for <18% of observations. BERTrand architecture can be easily adapted to CDR3*α*-annotated data in the future, when more such data are available. Due to the low MHC diversity in the data, the aspects of the interactions between the MHC and the TCR were also omitted. However, we are confident that the growing collective effort we are witnessing in this field will eventually lead to populating databases with large amounts of fully annotated data. We believe this will open new doors for a holistic solution to the pMHC: TCR binding problem, with profound consequences for the development of immune-related therapies.

## Supplementary Material

btad468_Supplementary_Data

## Data Availability

The data underlying this article are available in GitHub, at https://github.com/SFGLab/bertrand. The datasets were derived from sources in the public domain, outlined in Table 1. The complete results of model training and evaluation are available at https://drive.google.com/file/d/1U4lA9TsW0IQJXSk-7e478AAUU_59jNdV/view?usp=sharing
