# Supplementary material for: BERTrand—peptide:TCR binding prediction using Bidirectional Encoder Representations from Transformers augmented with random TCR pairing
Source: Bioinformatics. 2023 Aug 3;39(8):btad468. doi: 10.1093/bioinformatics/btad468 (PMC10444968; doi:10.1093/bioinformatics/btad468)
Supplement: btad468_Supplementary_Data [file btad468_supplementary_data.pdf]

Supplementary data

1 Peptide bias

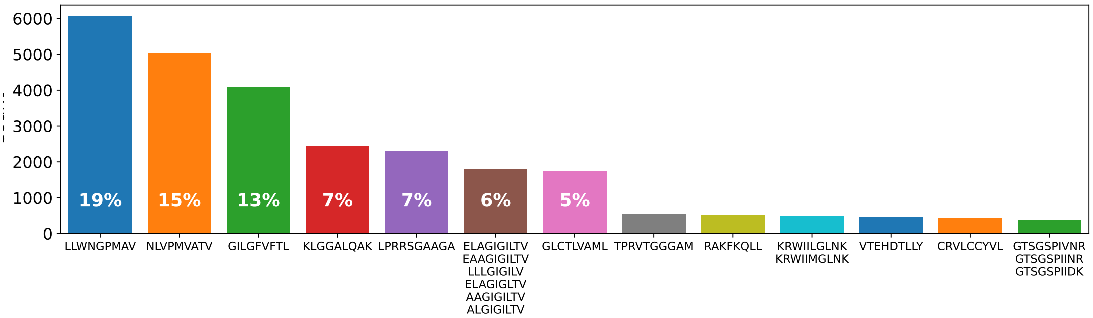

Figure 1: Number of binding TCRs for the most abundant peptide clusters. The fraction of observations for top 5 peptides is 61%. If mismatch pairing is applied, the model which learns to recognize the TCRs of these 5 peptides, could correctly classify 61% of negative examples in the dataset.

2 Outliers filtering

Adding reference TCRs introduces a potential problem in peptide:TCR binding prediction. Namely, reference TCRs that are out-of-distribution for the positive TCR sequences can be easily exploited by the model and classified as negative examples. This may result in AUROC inflation and incorrect results, as the model might guess the class from the sequence alone. The algorithm for outlier filtering is designed to minimize this risk by removing reference TCRs that can be easily classified as negatives. See Algorithm 3 below. Algorithms 1 for cross-val-score and 2 for cross-val-predict are presented for clarity, the standard implementations from scikit-learn were used.

Outliers filtering algorithm re-frames the outliers detection into a classification problem and uses an iterative approach to minimize the risk of easy negatives in the final dataset. Basically, we train a classifier to predict negativity based on TCR sequence alone and then remove a portion of reference TCRs with the highest predicted negativity. We repeat the process until AUROC of 0.5 is achieved.

Algorithm 1: cross_val_score - compute average AUROC in cross-validation for a classification problem

Input: X: sequences representation (onehot)
Input: y: target variable (1 for positive, 0 for reference)
Input: $N = 25$: number of splits
Input: $Model = XT\ (ExtremeTrees)$
Input: $Metric = AUROC$
Output: $score$: average cross-validated AUROC score

$scores \leftarrow []$
for $i \leftarrow 0$ **to** N **do**
  $X_{train}, y_{train}, X_{test}, y_{test} \leftarrow stratified\_split(X, y)$
  $model \leftarrow XT(X_{train}, y_{train})$
  $\hat{y}_{test} \leftarrow predict(model, X_{test})$
  $score \leftarrow AUROC(y_{test}, \hat{y}_{test})$
  $scores.append(score)$
return $mean(scores)$

Algorithm 2: cross_val_predict - averaged out-of-fold predictions for every observation

Input: X: sequences representation (onehot)
Input: y: target variable (1 for positive, 0 for reference)
Input: $K_{folds} = 5$: number of folds
Input: $N_{repeats} = 10$: number of repetitions
Input: $Model = XT\ (ExtremeTrees)$
Output: $predictions$: average cross-validated predictions for every observation
$X \leftarrow concat(positives, negatives)$
$y \leftarrow 1\ if\ x\ \in\ positives\ else\ 0\ \forall x \in X$
$predictions \leftarrow []$
for $i \leftarrow 0$ **to** $N_{repeats}$ **do**
  for do
    $fold\_predictions \leftarrow []$
    $X_{train}, y_{train}, X_{test}, y_{test} \leftarrow stratified\_K\_fold(X, y, K = K_{folds})$
    $model \leftarrow XT(X_{train}, y_{train})$
    $\hat{y}_{test} \leftarrow predict(model, X_{test})$
    $fold\_predictions.append(\hat{y}_{test})$
  $\hat{y} \leftarrow concat(fold\_predictions)$
  $predictions.append(\hat{y})$
return $mean(predictions)$

Algorithm 3: Outliers filtering

Input: X^+: binding TCRs
Input: X^-: reference TCRs
Input: $N = 2000$: number of outliers removed during one iteration
Output: Curated set of reference TCRs that aren't easily distinguishable from positive TCRs
1 $X^- \leftarrow X^- \setminus \{\text{CDR3β}_i \in X^- \mid \min_{\forall CDR3\beta_j \in X^+} Levenshtein(\text{CDR3β}_i, \text{CDR3β}_j) \leq 1\}$
2 $X \leftarrow concat(X^+, X^-)$
3 $y \leftarrow 1\ if\ x\ \in\ X^+\ else\ 0\ \forall x \in X$
4 **while** $cross\_val\_score(X, y) > 0.5$ **do**
5   $\hat{y} \leftarrow cross\_val\_predict(X, y)$
6   $threshold \leftarrow sort(\hat{y})[N]$
7   $X^-_{easy}, y^-_{easy} \leftarrow \{X_i, y_i \in X, y \mid y_i = 0 \quad \& \quad \hat{y_i} < threshold\}$
8   $X, y \leftarrow (X, y) \setminus X^-_{easy}, y^-_{easy}$
return $X[y = 0]$

First, we remove reference TCRs identical or differing by 1 amino acid from the set of positive TCR (line 1 in Algorithm 3). Then, we construct a dataset of positive TCRs and reference TCRs as potential negatives (lines 2-3). We then iteratively check AUROC, and if it is above 0.5 (line 4), an iteration of easy negatives removal is performed. We obtain average out-of-fold predictions for every observation (line 5) and then remove first 2000 observations sorted by predicted negativity (lines 6-8).

In our study, we use one-hot encoding and Extreme trees classifier due to speed concerns. It took 119 iterations of easy negatives filtering to remove outliers from a set of around 580k of reference TCRs, each step performing 25 model training and evaluation episodes in cross_val_score and another 50 in cross_val_predict, which is 8925 in total. The algorithm ran on a computer with 128 CPUs for 6 days and the reference TCRs dataset was reduced to around 110k. The training time of Extreme trees on one-hot features was 30-60 seconds on average, reducing as the number of observations decreased. We acknowledge the disadvantages of using such a simple model and not BERTrand itself for this task, but the computational cost made it unattainable for us.

3 NLP pre-training

The hypothetical peptide:TCR repertoire for pre-training was created by matching presented peptides from mass spectrometry experiments with reference CDR3β sequences. Around 11M reference TCRs from healthy donors' repertoires were used, 9M were used for training and 2M for validation. Masked language modelling (MLM) pre-training of the BERT neural network was performed. The effects of MLM pre-training can be seen in Figure 2.

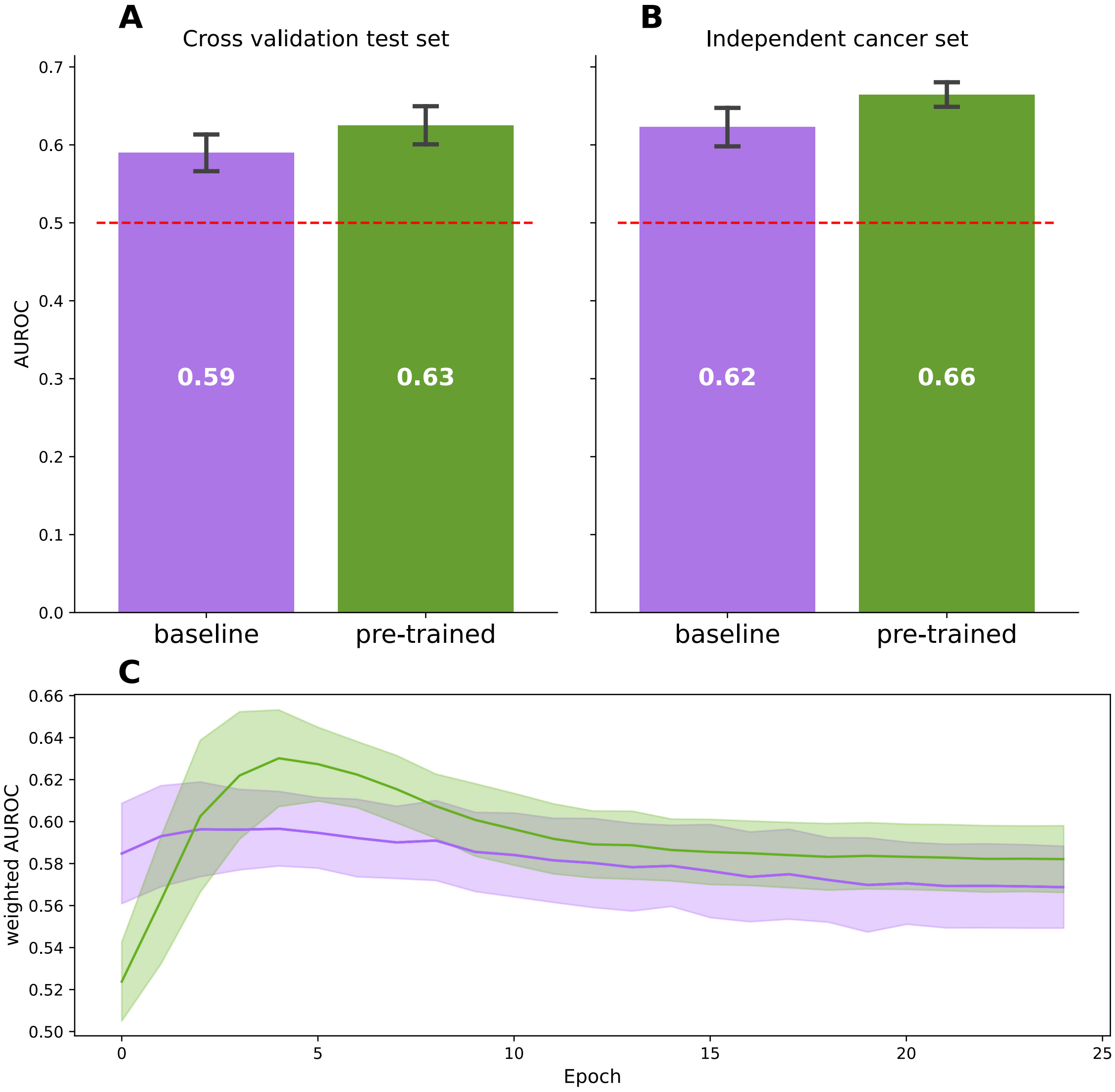

Figure 2: A comparison of pre-trained BERT and BERT with random weights for peptide:TCR binding prediction (A) average AUROC in cross-validation (B) average AUROC on the independent cancer set (C) weighted average AUROC on the early stopping set by epochs. The pre-trained model achieves significantly better results and overfits less during training.

4 Hyperparameters

Below are network hyperparameters of BERTrand. Note that the MLM token classification head is omitted for brevity.

```
BERTrand(
  (bert): BertModel(
    (embeddings): BertEmbeddings(
      (word_embeddings): Embedding(25, 512, padding_idx=0)
      (position_embeddings): Embedding(64, 512)
      (token_type_embeddings): Embedding(2, 512)
      (LayerNorm): LayerNorm((512,), eps=1e-12, elementwise_affine=True)
      (dropout): Dropout(p=0.1, inplace=False)
    )
    (encoder): BertEncoder(
      (layer): ModuleList(
        (0): BertLayer(
          (attention): BertAttention(
            (self): BertSelfAttention(
              (query): Linear(in_features=512, out_features=512, bias=True)
              (key): Linear(in_features=512, out_features=512, bias=True)
              (value): Linear(in_features=512, out_features=512, bias=True)
              (dropout): Dropout(p=0.1, inplace=False)
            )
            (output): BertSelfOutput(
              (dense): Linear(in_features=512, out_features=512, bias=True)
              (LayerNorm): LayerNorm((512,), eps=1e-12, elementwise_affine=True)
              (dropout): Dropout(p=0.1, inplace=False)
            )
          )
          (intermediate): BertIntermediate(
            (dense): Linear(in_features=512, out_features=2048, bias=True)
          )
          (output): BertOutput(
            (dense): Linear(in_features=2048, out_features=512, bias=True)
            (LayerNorm): LayerNorm((512,), eps=1e-12, elementwise_affine=True)
            (dropout): Dropout(p=0.1, inplace=False)
          )
        )
        (1): BertLayer //same as above
        ...
        (7): BertLayer
     )
  )
  (dropout): Dropout(p=0.1, inplace=False)
  (classifier): ClassificationHead(
    (dense): Linear(in_features=512, out_features=512, bias=True)
    (dropout): Dropout(p=0.1, inplace=False)
    (out_proj): Linear(in_features=512, out_features=2, bias=True)
  )
)
```

Below are training hyperparameters of BERTrand for pre-training and fine-tuning.

	Pre-training	Fine-tuning
Loss	Categorical cross entropy	Focal loss (γ=3, α=0.25)
Optimizer	AdamW	
Batch size	128	32
Epochs	100	25
Learning rate	5×10^{-5}	10^{-5}
Learning rate warm-up steps	10000	5000
Weight decay	10^{-4}	10^{-5}
Sample weights	Uniform	See sample weight formula below

Table 1: Training hyperparameters of BERTrand

$$
\begin{aligned}
X, y &\leftarrow \{\text{peptide}^i, \text{CDR3}\beta^i\},\ \{y^i\} \text{ for } i = 1 \ldots n \\
cluster_{pep} &\leftarrow hclust(\{\text{peptide} \in X\}, distance = \text{Levenshtein}, linkage = \text{'single'}, cutoff = 3) \\
cluster_{tcr} &\leftarrow hclust(\{\text{CDR3}\beta \in X\}, distance = \text{Levenshtein}, linkage = \text{'complete'}, cutoff = 3) \\
C_{pep} &\leftarrow \{\ |\{\text{peptide}, \text{CDR3}\beta \in X \mid cluster_{pep}[\text{peptide}] = c\}| \text{ for } c \in cluster_{pep}\ \} \\
C_{tcr} &\leftarrow \{\ |\{\text{peptide}, \text{CDR3}\beta \in X \mid cluster_{tcr}[\text{CDR3}\beta] = c\}| \text{ for } c \in cluster_{tcr}\ \} \\
sample\ weight(\text{peptide}, \text{CDR3}\beta) &\leftarrow 10 \times \frac{1}{\log\left(2 + C_{pep}[cluster_{pep}[\text{peptide}]]\right)} \times \frac{1}{\log\left(2 + C_{tcr}[cluster_{tcr}[\text{CDR3}\beta]]\right)}
\end{aligned}
$$

Sample weight formula. Each observation was weighted inversely to its peptide and TCR cluster counts (C_{pep} and C_{tcr}) The logarithmic function was used to prevent the weights from going close to zero for the most popular groups of observations. The product of inverse counts was multiplied by 10 for numerical stability.

5 Evaluation

The specifics of the evaluation procedure are outlined in Algorithm 4. It illustrates a single round of cross-validation. The inputs are the viral and cancer datasets, augmented with negative decoys. First, we perform the clustering of peptides and TCRs (lines 1 and 2). (In practice, the clustering is done once before evaluation and is not repeated for every single round). We count the number of positive observations for each peptide cluster (line 3). We also define the peptide and TCR clusters of the cancer dataset (lines 4 and 5). Next, we do balanced sampling of the peptide clusters for the test set (line 10). The sampling is done among the peptide clusters from the viral dataset (line 6): 2 clusters with over 1000 observations (line 7), 5 clusters with over 100 observations (line 8) and 7 clusters with over 5 observations (line 9), 14 peptide clusters in total. The test set is the observations from the viral dataset where the peptide belongs to one of the above 14 peptide clusters, excluding any peptide:TCR pairs where the peptide belongs to any peptide cluster in the cancer dataset (in practice, viral and cancer don't cluster together), as well as peptide:TCR pairs where the TCR belong to any TCR cluster in the cancer dataset (line 11). This increases the independence between each test set and the cancer set. Next, we define the TCR clusters of the test set (line 12), so that observations belonging to these clusters can be excluded from the training set. The training set is based on the viral dataset, excluding peptide and TCR clusters from cancer and test sets (line 13). Next, the model is trained on the training set (line 14). We obtain the predictions for the test set (line 15). P_{test} in line 16 is a vector of peptides and $supp\ P_{test}$ is a set of unique values in that vector. Test AUROC is computed per peptide and averaged (line 17). Cancer AUROC is computed in a similar way (lines 18-20).

Algorithm 4: Evaluation (single round)

Input: $X_{viral}, y_{viral} = \{\text{peptide}^i, \text{CDR3}\beta^i\},\ \{y^i\}$ for $i = 1 \dots n_{viral}$: viral dataset
Input: $X_{cancer}, y_{cancer} = \{\text{peptide}^i, \text{CDR3}\beta^i\},\ \{y^i\}$ for $i = 1 \dots n_{cancer}$: cancer dataset
Input: $model$: machine learning algorithm
Output: average AUROC for the cross-validation test set
Output: average AUROC for the independent cancer set

$cluster_{pep} \leftarrow hclust(\{\text{peptide} \in X_{viral} \cup X_{cancer}\},$
$\quad distance = \text{Levenshtein}, linkage = \text{'single'}, cutoff = 3)$
$cluster_{tcr} \leftarrow hclust(\{\text{CDR3}\beta \in X_{viral} \cup X_{cancer}\},$
$\quad distance = \text{Levenshtein}, linkage = \text{'complete'}, cutoff = 3)$
$C^+_{pep} \leftarrow \{\ |\{\text{peptide}^i, \text{CDR3}\beta^i \in X_{viral} \mid y^i = 1 \text{ and } cluster_{pep}[\text{peptide}] = c\}| \text{ for } c \in cluster_{pep}\ \}$
$pep\_clusters_{cancer} \leftarrow \{cluster_{pep}[\text{peptide}] \mid \text{peptide} \in X_{cancer}\}$
$tcr\_clusters_{cancer} \leftarrow \{cluster_{tcr}[\text{CDR3}\beta] \mid \text{CDR3}\beta \in X_{cancer}\}$
$pep\_clusters_{viral} \leftarrow \{cluster_{pep}[\text{peptide}] \mid \text{peptide} \in X_{viral}\}$
$pep\_clusters_{high} \leftarrow \{c \in pep\_clusters_{viral} \mid C^+_{pep}[c] >= 1000\}$
$pep\_clusters_{medium} \leftarrow \{c \in pep\_clusters_{viral} \mid 100 <= C^+_{pep}[c] <= 1000\}$
$pep\_clusters_{low} \leftarrow \{c \in pep\_clusters_{viral} \mid 5 <= C^+_{pep}[c] <= 100\}$
$pep\_clusters_{test} \leftarrow sample(pep\_clusters_{high}, 2) \cup$
$\quad sample(pep\_clusters_{medium}, 5) \cup$
$\quad sample(pep\_clusters_{low}, 7)$
$X_{test}, y_{test} \leftarrow \{\text{peptide}^i, \text{CDR3}\beta^i \in X_{viral}\}, \{y^i \in y_{viral}\} \mid$
$\quad cluster_{pep}[\text{peptide}^i] \in pep\_clusters_{test}$ and
$\quad cluster_{pep}[\text{peptide}^i] \notin pep\_clusters_{cancer}$ and
$\quad cluster_{pep}[\text{CDR3}\beta^i] \notin tcr\_clusters_{cancer}$
$tcr\_clusters_{test} \leftarrow \{cluster_{tcr}[\text{CDR3}\beta] \mid \text{CDR3}\beta \in X_{test}\}$
$X_{train}, y_{train} = \{\text{peptide}^i, \text{CDR3}\beta^i \in X_{viral}\}, \{y^i \in y_{viral}\} \mid$
$\quad cluster_{pep}[\text{peptide}^i] \notin pep\_clusters_{test} \cup pep\_clusters_{cancer}$ and
$\quad cluster_{pep}[\text{CDR3}\beta^i] \notin tcr\_clusters_{test} \cup tcr\_clusters_{cancer}$
$train(model, X_{train}, y_{train})$
$\hat{y}_{test} = predict(model, X_{test})$
$P_{test} \leftarrow [\text{peptide} \in X_{test}]$
$score_{test} = \frac{1}{|supp\ Ptest|} \sum_{p \in supp\ P_{test}} AUROC(y_{test}[P_{test} = p], \hat{y}_{test}[P_{test} = p])$
$\hat{y}_{cancer} = predict(model, X_{cancer})$
$P_{cancer} \leftarrow [\text{peptide} \in X_{cancer}]$
$score_{cancer} = \frac{1}{|supp\ P_{cancer}|} \sum_{p \in supp\ P_{cancer}} AUROC(y_{cancer}[P_{cancer} = p], \hat{y}_{cancer}[P_{cancer} = p])$
return $score_{test},\ score_{cancer}$

6 Baseline estimation

During evaluation, we considered two baselines for peptide-TCR binding prediction (see Table 2). The first baseline is the random model. For the AUROC metric it is equal to 0.5. Average precision (AP) has no constant random baseline as AUROC does, instead the baseline AP level for a dataset is the percent of positive observations. The difference in AP baselines between outliers filtering and basic filtering experiments comes from the ratio of negative decoy observations. In tests with basic filtering there were more reference TCRs available, so the number of negatives was 4 to 1 (AP baseline is equal to 0.2). In tests with the outliers filtering the ratio was lower - 3 to 1 (AP baseline is equal to 0.25).

The second baseline is based on the predictivity of the TCR sequence without the peptide sequence. As mentioned in the Outliers filtering section, we used a surrogate model (Extra Trees classifier) in an attempt to remove out-of-distribution reference TCRs. However, our model architecture is more sophisticated and BERTrand may exploit the biases in the TCR sequences that the outliers filtering procedure overlooked. We trained BERTrand with the same exact setup, but the peptide input was an empty string. As you can see

in Table 2, these empirical baselines are higher than the random ones. For experiments without outliers filtering, they are expectedly very high, which demonstrates the importance of this step in the pipeline.

		Cross-validation test set		Independent cancer set	
Baseline	Experiment	AUROC	AP	AUROC	AP
Random	Outliers filtering	0.5	0.25	0.5	0.25
TCR sequence only	Outliers filtering	0.55 ± 0.03	0.31 ± 0.03	0.58 ± 0.04	0.34 ± 0.04
Random	Basic filtering	0.5	0.2	0.5	0.2
TCR sequence only	Basic filtering	0.64 ± 0.01	0.34 ± 0.02	0.68 ± 0.01	0.39 ± 0.03

Table 2: Baseline values for all metrics and subsets

7 Average precision

We did additional validation using a different metric. It can be argued that the area under ROC curve (AUROC) is not the best metric for the problem - the low probability of binding across the peptide:TCR distribution might call for the metric that prioritizes specificity over sensitivity. One such metric is average precision (AP), which is the area under the precision-recall curve. This metric disregards the true negatives and instead focuses on the ranking of positive examples. In order to additionally verify our results, we calculated the average precision for BERTrand and 4 benchmarks. They are illustrated in Figure 3.

AP is unaffected by the number of true negatives, which means the ranking of the positive examples by BERTrand is very similar in both of these tests. However, achieving 0.55 over 0.34 TCR only baseline is objectively better than 0.55 over a 0.39 TCR only baseline. Also, other 4 methods do achieve inflated results in AP without the outliers filtering. Thus we argue that there is still merit in doing the outliers filtering, even under the AP metric. Nevertheless, the AP metric confirms that BERTrand outperforms the benchmarks in the task of peptide:TCR binding prediction.

Although AP prioritizes specificity over sensitivity, this may not always be a desirable behaviour for the model in a real world scenario. Consider the application of BERTrand between rounds of peptide library optimization for a given TCR (e.g. through phage display experiments). BERTrand can be used to rank the potential binders and reduce the number of peptides tested *in vitro*. The probability of binding between the TCR and peptides in the library after several rounds of optimization may be higher than the 10^{-4} reported in the literature, and the number of true negatives in the ranking produced by the model may start to matter. We believe that AUROC is a more universal metric, so AP is a secondary metric in this study.

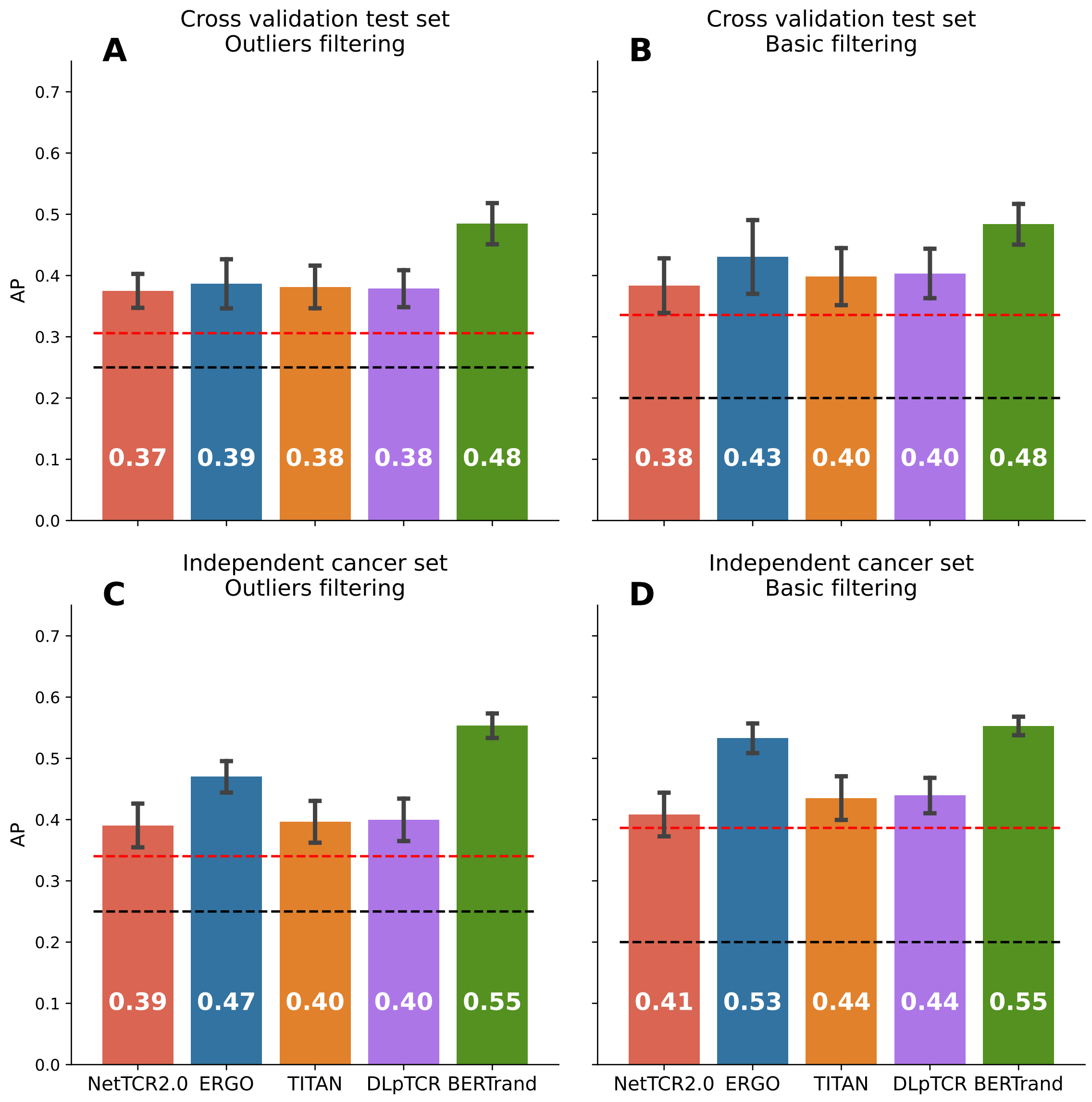

Figure 3: Benchmarks with AP. (A) Results for the cross-validation test set. (B) Results for the cross validation test set without outliers filtering. (C) Results for the independent cancer set. (D) Results for the independent cancer set without outliers filtering.
